# Assessing the Efficacy of a Brief Universal Family Skills Program on Child Behaviour and Family Functioning in Families in Gilgit Baltistan, Pakistan

**DOI:** 10.1192/bjo.2024.125

**Published:** 2024-08-01

**Authors:** Muqaddas Asif, Aala El-Khani, Nusrat Husain, Nasim Chaudhry, Imran B. Chaudhry

**Affiliations:** 1 Pakistan Institute of Living and Learning, Karachi, Pakistan; 2 University of Manchester, Manchester, United Kingdom; 3 United Nations Office on Drugs and Crime, Vienna, Austria; 4 Ziauddin University, Karachi, Pakistan

## Abstract

**Aims:**

The burden of mental health difficulties is a global problem and preventing them from childhood is paramount. Children living in challenged and underserved settings can suffer various harmful lifelong consequences including alcohol and substance abuse, low self-esteem, health issues, poor school performance, self-harm and suicide. This study aims to assess the feasibility, acceptability and efficacy of the culturally adapted Strong Families program in improving child behaviour and family functioning in families living in a challenged setting i.e. Gilgit-Baltistan (GB), Pakistan.

**Methods:**

This is a two-arm, multisite feasibility randomised controlled trial with 90 families (n = 45 in intervention, and n = 45 in waitlist group) including a female primary caregiver (mostly mother) and at least one of their children between the age of 8–15 years in three districts of GB. There will be three raters’ blind assessments: at baseline, week 2, and 6 weeks after Strong Families Program sessions.

**Results:**

Strong Families Program is a brief evidence-based prevention programme designed to improve parenting skills, child well-being and family mental health. The primary outcome measures include the feasibility of Strong Families, as determined by families' recruitment, attendance rates, and program completeness (mean number of sessions attended, attrition rates). Additionally, purposefully selected participants, including up to 5 caregivers from each study site, researchers, and facilitators delivering the intervention, will be interviewed. Descriptive statistics will be used to analyse primary and secondary outcomes. The process evaluation will be conducted in terms of program context, reach, fidelity, dose delivered and received, implementation, and recruitment.

**Conclusion:**

The findings from this feasibility trial hold the potential to carry out the large multicentre trial of clinical and cost-effectiveness and scale-up across Pakistan and other similar settings to meaningfully impact child behaviour and family dynamics in culturally diverse contexts.

